# Uncovering the Early Assembly Mechanism for Amyloidogenic β_2_-Microglobulin Using Cross-linking and Native Mass Spectrometry*

**DOI:** 10.1074/jbc.M115.691063

**Published:** 2015-12-10

**Authors:** Zoe Hall, Carla Schmidt, Argyris Politis

**Affiliations:** From the ‡ Department of Biochemistry, University of Cambridge, 80 Tennis Court Road, Cambridge CB2 1GA, United Kingdom,; § Department of Chemistry, University of Oxford, South Parks Road, Oxford OX1 3QZ, United Kingdom, and; ¶ Department of Chemistry, King's College London, 7 Trinity Street, London SE1 1DB, United Kingdom

**Keywords:** amyloid, mass spectrometry (MS), molecular modeling, protein cross-linking, structural model, mass spectrometry

## Abstract

β_2_-Microglobulin (β_2_m), a key component of the major histocompatibility class I complex, can aggregate into fibrils with severe clinical consequences. As such, investigating the structural aspects of the formation of oligomeric intermediates of β_2_m and their subsequent progression toward fibrillar aggregates is of great importance. However, β_2_m aggregates are challenging targets in structural biology, primarily due to their inherent transient and heterogeneous nature. Here we study the oligomeric distributions and structures of the early intermediates of amyloidogenic β_2_m and its truncated variant ΔN6-β_2_m. We established compact oligomers for both variants by integrating advanced mass spectrometric techniques with available electron microscopy maps and atomic level structures from NMR spectroscopy and x-ray crystallography. Our results revealed a stepwise assembly mechanism by monomer addition and domain swapping for the oligomeric species of ΔN6-β_2_m. The observed structural similarity and common oligomerization pathway between the two variants is likely to enable ΔN6-β_2_m to cross-seed β_2_m fibrillation and allow the formation of mixed fibrils. We further determined the key subunit interactions in ΔN6-β_2_m tetramer, revealing the importance of a domain-swapped hinge region for formation of higher order oligomers. Overall, we deliver new mechanistic insights into β_2_m aggregation, paving the way for future studies on the mechanisms and cause of amyloid fibrillation.

## Introduction

The major histocompatibility class I complex (MHC I) is found on the cell surface of all nucleated cells and is responsible for antigen presentation (1, 2). β_2_-Microglobulin (β_2_m)^2^ is a key component of this complex. After its dissociation from MHC I, serum β_2_m is broken down in the kidney. The buildup of circulating β_2_m can result as a consequence of renal dysfunction and long term hemodialysis. This leads to amyloid fibril deposition in osteoarticular tissues and joint destruction in a condition known as dialysis-related amyloidosis.

Amyloidogenic proteins such as β_2_m tend to self-assemble into higher order oligomeric species through a complex aggregation process, which can ultimately lead to the formation of fibrils (3–6). Despite the clinical significance of amyloid formation, the principles governing the mechanisms for the aggregation process of β_2_m and related proteins remains largely unknown, primarily due to the transient nature of intermediates on-pathway to fibril formation (7). Structural elucidation of β_2_m oligomeric intermediates is, therefore, a challenging task that is further complicated by the uncertainty in differentiating between specific and nonspecific protein aggregates.

Despite these obstacles progress has been made most prominently from techniques such as nuclear magnetic resonance spectroscopy (NMR) (8), x-ray crystallography (9), atomic force microscopy (10), cryo-electron microscopy (EM) (11), and hydrogen/deuterium exchange (12) and by combining NMR with mass spectrometry (MS) (13). MS in particular is well suited for studying heterogeneous assembly intermediates, including proteins populating multiple oligomeric states (14). When coupled with ion mobility (IM), IM-MS allows the separation of different conformational states of co-populated oligomers (5, 15). IM-MS has been successfully employed to investigate the structure and dynamics of amyloid assembly intermediates revealing information on the aggregation process of Aβ40 and Aβ42 complexes (15, 16) and more recently human amylin (17). Structural insights have also been gained for β_2_m (18, 19) where IM-MS experiments suggested either an elongated or more compact assembly mechanism for full-length β_2_m under different solution conditions (20).

The integration of different experimental techniques with modeling can provide powerful means to interrogate candidate models of protein assemblies (21–25). In particular so-called hybrid approaches, which combine information from complementary experiments, have shed light on complexes intractable by single techniques, exemplified by the structural elucidation of the nuclear pore complex (26), the 26S proteasome (27, 28), and the eukaryotic translation initiation factor 3 (29, 30).

Here we used an integrative MS-based strategy for generating three-dimensional structural models of the oligomeric assembly intermediates of ΔN6-β_2_m, a truncated β_2_m isoform (11.1-kDa monomer). ΔN6-β_2_m makes up to 30% of amyloid deposits extracted from dialysis-related amyloidosis patients and can act as a seed for full-length β_2_m fibrillogenesis *in vitro* (1). Contrary to full-length β_2_m, ΔN6-β_2_m is highly amyloidogenic at neutral pH, making it a convenient model for studying β_2_m aggregation under laboratory conditions (2). Furthermore, the x-ray crystal structure of the dimeric intermediate built by the self-association of two ΔN6-β_2_m monomers was recently solved, and proposed as a building block for growing oligomers on-pathway to fibril formation (1). In this structure domain-swapping occurs through the so-called hinge region, which corresponds to two NHVTLSQ heptapeptides interacting in an antiparallel fashion (1).

Using a combination of experimental and computational techniques, we predict the structures and an early assembly mechanism for ΔN6-β_2_m oligomers. We further compare oligomers of the truncated variant with those of the full-length protein, highlighting similar oligomeric distributions and compact topologies as well as inter- and intraprotein distances. This points to a common assembly mechanism in the early stages of their aggregation and may facilitate the ability of the truncated variant to cross-seed and form mixed fibrils (31) with full-length β_2_m *in vivo*. The data and the structural models generated from the integrative strategy further suggest an elongation mechanism of monomer addition consistent with domain swapping and self-templated growth. Furthermore, our model for ΔN6-β_2_m tetramer shows that the domain-swapped hinge region found in ΔN6-β_2_m dimer is key to both intra- and interdimer interactions.

## Experimental Procedures

### 

#### 

##### Protein Preparation

ΔN6-β_2_m and β_2_m were expressed in *Escherichia coli* and purified using ion exchange chromatography and size exclusion chromatography as previously described (1). Lyophilized protein was dissolved in 100 mm ammonium acetate, pH 5, before MS analysis.

##### Ion Mobility-Mass Spectrometry

IM-MS experiments were performed on a quadrupole ion mobility time-of-flight mass spectrometer (Synapt HDMS, Waters Corp., Manchester, UK) modified such that the traveling-wave IM cell is replaced with an 18-cm drift cell with radial RF confinement (RF amplitude 200 V) and a linear voltage gradient along the axis of ion transmission, as described in detail previously (32). The following parameters were used: source pressure 4–6 mbar, capillary voltage 1.0–1.5 kV, sample cone voltage 20 V, bias voltage 20 V, IM entrance DC 5 V, trap gas 6 ml min^−1^, trap collision energy 5 V. Helium (2 torr) was used as the buffer gas, and the drift voltage varied from 50 to 200 V. All spectra were mass-calibrated using cesium iodide (100 mg ml^−1^).

##### Chemical Cross-linking MS

50 μl of ΔN6-β_2_m or β_2_m were cross-linked with 10 μl of a 25 mm 1:1 mixture of deuterated (d4) and non-deuterated (d0) bis[sulfosuccinimidyl] suberate (BS3). The reaction mixture was incubated at 25 °C and 400 rpm for 1 h. 10 μl of the cross-linked proteins were analyzed by gel electrophoresis (NuPAGE system, Invitrogen) according to the manufacturer's protocol. The proteins were digested in-gel as described elsewhere (33).

The mixture of cross-linked and non-cross-linked peptides was analyzed by liquid chromatography-coupled tandem-mass spectrometry (LC-MS/MS) employing an LTQ-Orbitrap XL hybrid mass spectrometer (Thermo Scientific) coupled with a Dionex UltiMate 3000 RSLC nano System (Thermo Scientific).

The peptides were separated by nanoLC (mobile phase A, 0.1% (v/v) formic acid; mobile phase B, 80% (v/v) acetonitrile/0.1% (v/v) formic acid). The peptides were loaded onto a trap column (2 cm, HPLC column Acclaim PepMap 100, C18, 100-μm inner diameter, particle size 5 μm; Thermo Scientific) and separated on an analytical C18 capillary column (50 cm, HPLC column Acclaim PepMap 100, C18, 75-μm inner diameter particle size 3 μm; Thermo Scientific) at a flow rate of 300 nl/min and a gradient of 5–80% (v/v) mobile phase B over 74 min.

The peptides were directly eluted into the mass spectrometer. Mass spectrometric conditions were: spray voltage of 1.8 kV, capillary temperature 180 °C, normalized collision energy 35% at an activation of *q* = 0.25, and an activation time of 30 ms. The LTQ-Orbitrap XL was operated in data-dependent mode. Survey full scan MS spectra were acquired in the orbitrap (*m*/*z* 300–2,000) with a resolution of 30,000 at *m*/*z* 400 and an automatic gain control target at 10^6^. The five most intense ions were selected for collision-induced dissociation in the linear ion trap at an automatic gain control target of 30,000. Selection of previously selected precursor ions was dynamically excluded for 30 s. Singly charged ions as well as ions with unrecognized charge state were also excluded. Internal calibration of the Orbitrap was performed using the lock mass option (lock mass: *m*/*z* 445.120025) (34).

mzXML files were generated from raw data using the MassMatrix file conversion tool. Potential cross-links were identified by searching against a reduced database containing ΔN6-β_2_m and β_2_m protein sequences using the MassMatrix Database Search Engine (35). Search parameters were: enzyme, trypsin; missed cleavage sites, two; variable modifications, carbamidomethylation of cysteines and oxidation of methionine; mass accuracy filter, 10 ppm for precursor ions and 0.8 Da for fragment ions; minimum pp and pp2 values, 5.0; minimum pptag, 1.3; maximum number of cross-links per peptide, 1. All searches were performed twice, including the deuterated and the non-deuterated BS3 cross-linker, respectively.

##### Modeling Restraints from Cross-linking

To test if the identified cross-links in the ΔN6-β_2_m tetramer (or trimer) were arising from interdimer or intradimer interactions, we projected the cross-links confirmed by MS/MS quality onto the dimer x-ray crystal structure (PDB ID 2X89). We measured the physical Cα-Cα distances (36) to check if these were within the upper-bound interresidue distance threshold (35 Å) (37). The cross-links, which do not satisfy our distance threshold, are more likely to generate from interdimer interactions in the tetramer (or trimer) and were, therefore, assigned as such in our modeling analysis. Those that were within the distance threshold were most likely to arise from intradimer interactions.

##### MS-based Hybrid Approach

**FIGURE 1.**
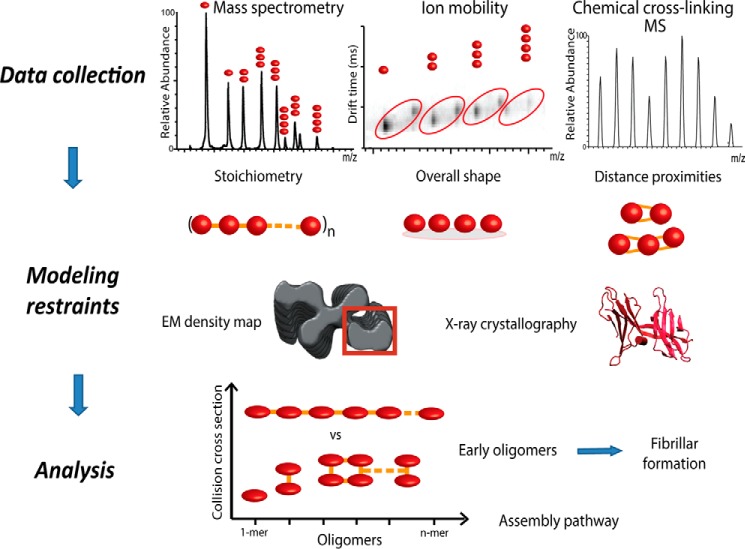
**Integrative modeling workflow for structure characterization of oligomeric protein assemblies.** To predict the structures of higher oligomeric states of proteins, we implement an integrative modeling strategy. Here we use information derived from native MS, IM-MS, and CX-MS. The acquired data and other available information are converted into spatial restraints, which are subsequently utilized by a scoring function to guide the search for candidate model structures. Finally, an analysis step (*e.g.* clustering) of the top-scoring models determined the most likely structures of the oligomeric assembly pathways.

We employed a hybrid approach for structural determination of oligomeric intermediates of ΔN6-β_2_m, primarily based on native MS, IM-MS and cross-linking MS (CX-MS) (27, 29) (Fig. 1). From native MS, we established the oligomeric state of the identified complexes (38). By combining MS with IM, topological information in the form of an orientation-averaged collision cross-section (CCS) was derived (39). The measured CCS from IM-MS was used as shape restraint for interrogating candidate structural models (22). CX-MS identified lysines in close proximity and was used as a distance restraint (27). In addition to MS data, we made use of available structures from x-ray crystallography and NMR as well as EM density maps. The atomic level structures were used as starting points in our modeling strategy, whereas a segment of the EM map of β_2_m assembled into fibrils (EM Database ID 1613; type A) was used as a volume restraint, as there is no available EM density map for ΔN6-β_2_m fibrils (11). Structural information obtained from these methods was encoded into restraints and exploited by a scoring function for subsequent modeling analysis.

##### Integrative Modeling and Scoring Function

We generated structural models of the assemblies by employing a Monte Carlo search algorithm. The building process was guided by a scoring function that encoded the experimental data as a sum of individual restraints. This scoring function (*S*) evaluates the ensemble of candidate models generated against their quality-of-fit with the input data.

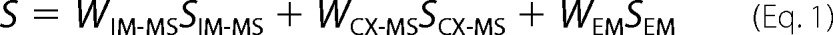
 where *S*_IM-MS_ and *S*_CX-MS_ refer to IM-MS and CX-MS restraints, respectively. *S*_IM-MS_ was implemented as a harmonic potential function (22, 23), whereas *S*_CX-MS_ was applied as a distance restraint between two interacting residues (27, 29). The EM (*S*_EM_) restraint assessed the quality-of-fit between the model and the corresponding molecular volume of an appropriate section of the EM map, as defined by the cross-correlation coefficient. IM-MS, CX-MS, and EM were given 1:2:2 weightings (*W*), respectively, consistent with previous benchmark studies (27).

To assess the uniqueness of the ensemble of generated models we performed ensemble analysis (*e.g.* clustering of top-scoring solutions), and the final solution was selected from the major cluster. The visual molecular dynamics (VMD) and the UCSF Chimera packages were used for visualization of the structures (40).

##### Collision Cross-section Calculations

To interpret the experimentally obtained CCS values, we compared them to theoretically calculated CCSs (41). Theoretical CCSs were obtained with the open source MOBCAL code using the projection approximation (PA) algorithm (42, 43). The PA method is known to underestimate the experimental CCS of proteins by neglecting multiple collisions between ions and buffer gas (7, 43). However, it has been shown that it is correlated with the experimental CCS for protein complexes (*R*^2^ > 0.99) (44). We use the scaled PA CCS as previously described, where the experimental CCS can be typically predicted (±3%) by multiplying the PA CCS by a factor of 1.14 (44). All CCS calculations include hydrogen atoms.

##### Molecular Dynamics Simulations

All simulations were performed in single (solution phase) or double floating-point precision (gas phase) with GROMACS 4.5.3 using the OPLS-AA/L forcefield (45).

Gas-phase simulations (10 ns) were performed at 300 K as described previously (46), The MS observed charge state was distributed evenly over solvent-accessible basic residues (<5 Å from the surface) (47, 48). As such, we assigned the charged residues for the 6+ monomer, 9+ dimer, 11+ trimer, and 13+ tetramer charge states of ΔN6-β_2_m. CCSs were calculated for structures every 25 ps using the scaled PA method implemented in MOBCAL.

Solution phase simulations (10 ns) were carried out similarly, except periodicity and a cutoff of 0.9 and 1.4 nm were used for electrostatic and van der Waals forces respectively; an integration step of 2 fs was used. Acidic and basic residues were charged as appropriate for solution. Total charge of the system was neutralized by the addition of an appropriate number of sodium ions.

##### Software and Scripts

Our integrative protocol was implemented within the open source Integrative Modeling Platform (IMP) software package.

## Results

### 

#### 

##### ΔN6-β_2_m Assembles into Compact Oligomers

**FIGURE 2.**
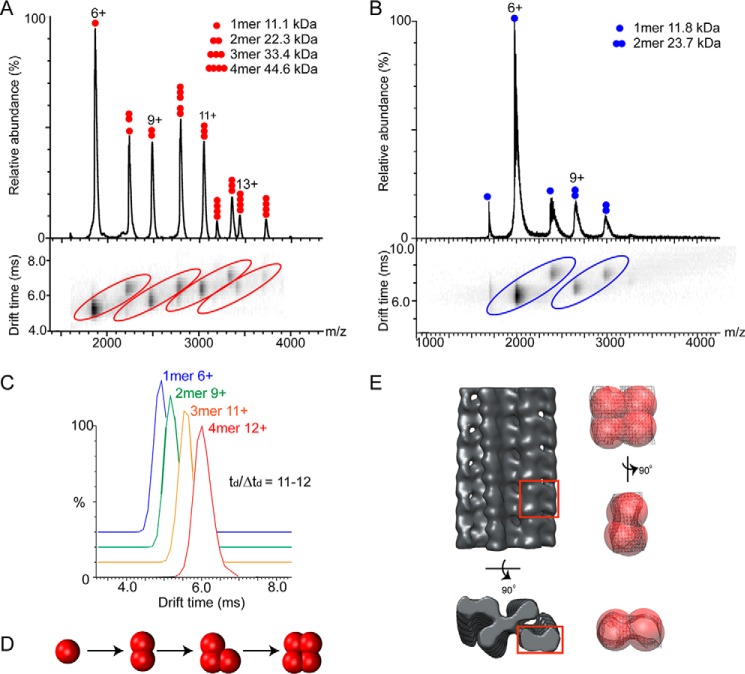
**IM-MS data and coarse-grained modeling reveal a compact tetrameric intermediate in ΔN6-β_2_m aggregation.** *A*, IM-MS of ΔN6-β_2_m (15 μm, pH 5) revealed four charge state distributions, corresponding to the 1-mer, 2-mer, 3-mer, and 4-mer. *B*, IM-MS of full-length β_2_m (15 μm, pH 5) reveals charge state distributions corresponding to 1-mer and 2-mer. Higher order oligomers up to tetramer could be observed at higher protein concentrations (30 μm, not shown). *C*, representative arrival time distributions for the 4-mer (12+), 3-mer (11+), 2-mer (9+), and 1-mer (6+) of ΔN6-β_2_m. *D*, the ΔN6-β_2_m tetramer was assembled from the monomer by the stepwise addition of subunits using a coarse-grained modeling strategy. The model with lowest score at each stage was chosen as the starting point for the subsequent addition of further monomer units. *E*, manual fitting of the low resolution ΔN6-β_2_m tetramer (best scored model after clustering) into the cryo-EM density map of β_2_m fibrils (EM Database ID 1613). Segments (*red box*) of the EM map are shown as *surface-and-mesh* representations in three different orientations.

We carried out IM-MS on ΔN6-β_2_m at various monomer concentrations (10–30 μm, pH 5) revealing multiple oligomeric species in equilibrium (Fig. 2*A*). Our experiments demonstrated that the formation of specific oligomers in solution is highly concentration-dependent (data not shown). At 10 μm, the predominant species observed were monomers and dimers, with a low amount of trimers formed. At 15 μm, we could clearly observe four charge state distributions corresponding to monomers, dimers, trimers, and tetramers of ΔN6-β_2_m (Fig. 2*A*). Higher concentrations (30 μm) revealed higher order oligomers (>pentamer). As a control we carried out similar experiments using cytochrome *c*, a monomeric protein of similar molecular mass (12 kDa). At 15 μm and below, only monomeric species were detected, whereas at higher concentrations (*e.g.* 30 μm), we could observe low intensity peaks for dimeric and trimeric species (data not shown). Therefore, we carried out experiments at a protein concentration of 15 μm to minimize any contribution from nonspecific aggregation (49). To correctly assign the oligomeric species of ΔN6-β_2_m, separation in mobility space was critical, as peaks at certain *m*/*z* values were co-populated with multiple species. For instance, *m*/*z* 2800 is composed of both dimer (8+ charge state) and trimer (12+ charge state) (Fig. 2*A*). Separation of different species with the same *m*/*z* but different CCS is a major strength of IM-MS.

CCS measurements were performed for all charge states of the four oligomers observed for ΔN6-β_2_m (15 μm). Arrival time distributions (ATD) had Gaussian peak shapes with *t*_D_/Δ*t*_D_ ∼ 11–12, consistent with a single conformer or group of closely related conformations (representative ATDs are shown in Fig. 2*C*) (39). The CCS deviation between different charge states of a particular oligomer was typically <5% (considered to be within experimental error), and we therefore averaged the CCS across all measured charge states. The average CCS for monomer (CCS_exp_, 1200 ± 36 Å^2^), dimer (CCS_exp_, 1900 ± 57 Å^2^), trimer (CCS_exp_, 2530 ± 76 Å^2^), and tetramer (CCS_exp_, 3057 ± 92 Å^2^) were measured (Table 1).

**TABLE 1:** **IM-MS experiments showed similar measured CCS for oligomeric species of ΔN6-β_2_m (15 μm, pH 5) and full-length β_2_m (15–30 μm, pH 5)**

Oligomeric species	ΔN6-β_2_m		β_2_m	
	CCS	Error	CCS	Error
	Å*^2^*		Å*^2^*	
Monomer	1200	36	1250	13
Dimer	1900	57	1994	20
Trimer	2530	76	2622*	52
Tetramer	3057	92	3213*	64

*^a^* CCS measurement at 30 μm.

We applied a similar approach to interrogate full-length β_2_m, which is expected to oligomerize to a lesser extent than its truncated variant at pH 5 (19). We observed monomer and dimer at a protein concentration of 15 μm (Fig. 2*B*), with trimers and tetramers only observed at higher concentrations (30 μm, data not shown). We measured CCSs for the oligomers of β_2_m at 15 μm (monomer and dimer) and at 30 μm (trimer and tetramer) (Table 1) revealing values <5% greater than those measured for ΔN6-β_2_m, consistent with the higher molecular weight. This suggests that the oligomers of full-length β_2_m and its truncated variant adopt similar conformations for their early oligomeric intermediates.

To establish the overall topology (*i.e.* compact or elongated) of ΔN6-β_2_m oligomers, we used the IM-MS data to inform a coarse-grained modeling strategy reported previously (22). Monomers were represented as spheres, with radii defined by the monomer CCS. Models were then built for the dimer and trimer by varying the intersubunit distances and angles. These models were then scored, and the one with the lowest total score at each stage was taken to the next step, to form the (*n*+*1*) oligomer (Fig. 2*D*). To sample conformational space for the tetramer, a Monte Carlo approach was used keeping the relative position of the three other subunits fixed. The 1% lowest scoring models were clustered, revealing three distinct clusters. The largest cluster (84.4%) is represented by a compact topological arrangement of subunits. This low resolution model of tetrameric ΔN6-β_2_m from IM-MS is consistent with a recently published EM map of β_2_m fibrils (Fig. 2*E*).

##### Protein Interactions in ΔN6-β_2_m Oligomers

**FIGURE 3.**
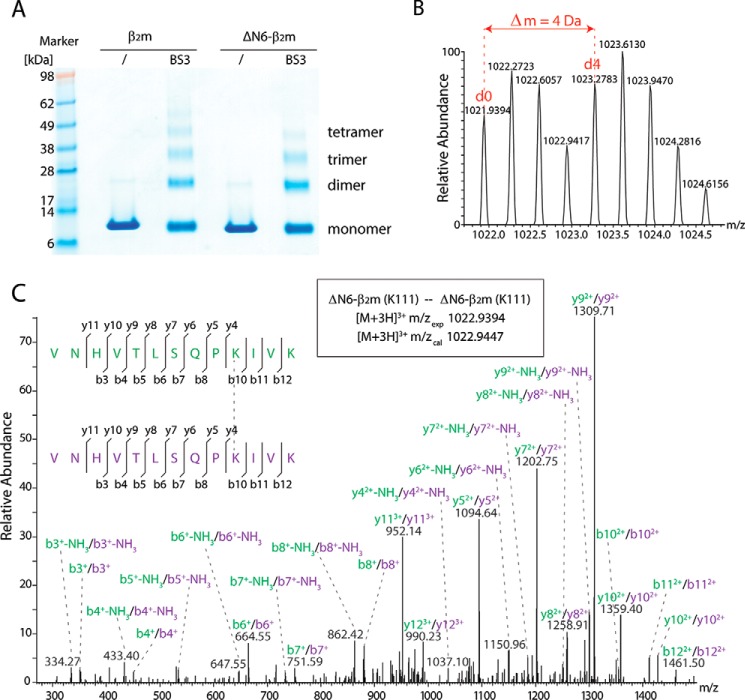
**Cross-linking of ΔN6-β_2_m and β_2_m.** *A*, ΔN6-β_2_m and β_2_m were cross-linked with a 1:1 mixture of BS3-*d*_0_/*d*_4_. The gels show the presence of monomers, dimers, trimers, and tetramers. *B*, cross-links were verified by the presence of peak pairs in the mass spectra corresponding to light (*d*_0_) and heavy (*d*_4_) cross-linked peptides. *C*, cross-linked dipeptides containing the same or overlapping peptide sequences are unambiguously assigned to interprotein cross-links. An example spectrum is shown.

Next, we employed CX-MS to gain insights into the interprotein interactions at atomic level. ΔN6-β_2_m was incubated with a 1:1 mixture of non-deuterated (*d*_0_) and deuterated (*d*_4_) BS3. The cross-linked proteins were separated by gel electrophoresis (Fig. 3*A*). The bands corresponding to monomer, dimer, trimer, and tetramer of ΔN6-β_2_m were cut, and the proteins were digested with trypsin. The mixture of peptides and cross-linked dipeptides for each band were analyzed by LC-MS/MS. In total, we obtained 138 potential hits after database searching, of which we validated 92 spectra by (i) the presence of peak pairs in the MS spectra (corresponding to BS3-*d*_0_ and *d*_4_; Fig. 3*B*) and (ii) by the quality of tandem-MS spectra, resulting in a false discovery rate of 33.33% (Table 2).

**TABLE 2:** **Identified cross-links for ΔN6-β_2_m and β_2_m oligomers**

Peptide sequence 1	Peptide sequence 2	Residue 1	Residue 2	No. spectra
**ΔN6-β_2_m monomer**				
VNHVTLSQP**K**IV**K**	**H_2N_-IQVYSR**	110	27	6
VNHVTLSQP**K**IV**K**WDRDM		110/113		64
VNHVTLSQP**K**IV**K**WDR		110/113		29

**ΔN6-β_2_m dimer**				
VNHVTLSQP**K**IV**K**	IV**K**WDR	110	113	2
VNHVTLSQP**K**IV**K**	IV**K**WDRDM	110	113	4
VNHVTLSQP**K**IV**K**	**H_2N_-IQVYSR**	110	27	4
VNHVTLSQP**K**IV**K**	VNHVTLSQP**K**IV**K**	110	110	13
IE**K**VEHSDLSFS**K**	IV**K**WDRDM	67	113	1
VNHVTLSQP**K**IV**K**WDR		110/113		40
VNHVTLSQP**K**IV**K**WDRDM		110/113		44
IE**K**VEHSDLSFS**K**DWSFYLLYYTEFTPTE**K**DEYACR		67/77		1

**ΔN6-β_2_m trimer**				
VNHVTLSQP**K**IV**K**WDR	IV**K**WDR	110	113	1
VNHVTLSQP**K**IV**K**WDR	IV**K**WDRDM	110	113	1
VNHVTLSQP**K**IV**K**	IV**K**WDR	110	113	2
VNHVTLSQP**K**IV**K**	IV**K**WDRDM	110	113	4
VNHVTLSQP**K**IV**K**	**H_2N_-IQVYSR**	110	27	6
IE**K**VEHSDLSFS**K**	VNHVTLSQP**K**IV**K**	67	110	2
IE**K**VEHSDLSFS**K**	IE**K**VEHSDLSFS**K**	67	67	1
VNHVTLSQP**K**IV**K**	VNHVTLSQP**K**IV**K**	110	110	9
VNHVTLSQP**K**IV**K**WDR		110/113		23
VNHVTLSQP**K**IV**K**WDRDM		110/113		24

**ΔN6-β_2_m tetramer**				
VNHVTLSQP**K**IV**K**	IV**K**WDR	110	113	2
VNHVTLSQP**K**IV**K**	IV**K**WDRDM	110	113	2
VNHVTLSQP**K**IV**K**	**H_2N_-IQVYSR**	110	27	2
IE**K**VEHSDLSFS**K**	VNHVTLSQP**K**IV**K**	67	110	2
VNHVTLSQP**K**IV**K**	VNHVTLSQP**K**IV**K**	110	110	7
VNHVTLSQP**K**IV**K**WDR		110/113		15
VNHVTLSQP**K**IV**K**WDRDM		110/113		15

**β_2_m monomer**				
VNHVTLSQP**K**IV**K**	TP**K**IQVYSR	110	26	84
VNHVTLSQP**K**IV**K**WDRDM	TP**K**IQVYSR	110	26	8
VNHVTLSQP**K**IV**K**WDR	TP**K**IQVYSR	110	26	3
VNHVTLSQP**K**IV**K**WDR	TP**K**IQVYSR	113	26	2
VNHVTLSQP**K**IV**K**WDR		110/113		40
VNHVTLSQP**K**IV**K**WDRDM		110/113		62

**β_2_m dimer**				
**H_2N_-IQRTPK**IQVYSR	TP**K**IQVYSR	21	26	3
IQRTP**K**IQVYSR	TP**K**IQVYSR	26	26	3
TP**K**IQVYSR	TP**K**IQVYSR	26	26	20
IE**K**VEHSDLSFS**K**	TP**K**IQVYSR	67	26	2
IE**K**VEHSDLSFS**K**	VNHVTLSQP**K**IV**K**	67	110	1
VNHVTLSQP**K**IV**K**	TP**K**IQVYSR	110	26	71
VNHVTLSQP**K**IV**K**	VNHVTLSQP**K**IV**K**	110	110	6
VNHVTLSQP**K**IV**K**	IV**K**WDRDM	110	113	1
VNHVTLSQP**K**IV**K**WDRDM	TP**K**IQVYSR	110	26	4
IV**K**WDRDM	TP**K**IQVYSR	113	26	1
VNHVTLSQP**K**IV**K**WDR	TP**K**IQVYSR	113	26	1
VNHVTLSQP**K**IV**K**WDR	TP**K**IQVYSR	113	26	3
VNHVTLSQP**K**IV**K**WDR		110/113		30
VNHVTLSQP**K**IV**K**WDRDM		110/113		41

**β_2_m trimer**				
VNHVTLSQP**K**IV**K**	TP**K**IQVYSR	110	26	50
VNHVTLSQP**K**IV**K**WDR	TP**K**IQVYSR	113	26	3
VNHVTLSQP**K**IV**K**WDRDM	TP**K**IQVYSR	110	26	2
TP**K**IQVYSR	TP**K**IQVYSR	26	26	9
IQRTP**K**IQVYSR	TP**K**IQVYSR	26	26	1
IE**K**VEHSDLSFS**K**	VNHVTLSQP**K**IV**K**	67	110	2
IE**K**VEHSDLSFS**K**	TP**K**IQVYSR	67	26	2
VNHVTLSQP**K**IV**K**	VNHVTLSQP**K**IV**K**	110	110	7
VNHVTLSQP**K**IV**K**WDR		110/113		16
VNHVTLSQP**K**IV**K**WDRDM		110/113		26

**β_2_m tetramer**				
VNHVTLSQP**K**IV**K**	TP**K**IQVYSR	110	26	30
TP**K**IQVYSR	TP**K**IQVYSR	26	26	8
IE**K**VEHSDLSFS**K**	TP**K**IQVYSR	67	26	4
VNHVTLSQP**K**IV**K**	VNHVTLSQP**K**IV**K**	110	110	5
VNHVTLSQP**K**IV**K**WDR		110/113		19
VNHVTLSQP**K**IV**K**WDRDM		110/113		25

Importantly we obtained dipeptides with the same or overlapping peptide sequences in the dimer, trimer, and tetramer bands, allowing their unambiguous classification as interprotein cross-links (Fig. 3*C*). The remaining cross-links are difficult to assign to intra- or interprotein cross-links, as the oligomers are formed by monomers with the same amino acid sequence, and their peptide sequences are not unique. We have, therefore, excluded these cross-links from our modeling and only implemented cross-links that were unique and unambiguously related to interprotein interactions.

To assess the structural differences between full-length β_2_m and its truncated form, we performed cross-linking experiments on β_2_m following the same strategy as above. Bands corresponding to monomer, dimer, trimer, and tetramer of β_2_m were cut, proteins were digested, and peptides were analyzed by LC-MS/MS. We obtained 529 potential hits after database searching and validated 322 of these manually (false discovery rate 39.13%). We obtained two unique cross-links from the monomer band and up to nine unique cross-links for the dimer, trimer, or tetramer bands (Table 2). These cross-links were in good agreement with those obtained for ΔN6-β_2_m (Table 2). Due to the longer amino acid sequence in β_2_m we identified additional interactions in the N-terminal regions of the protein. The high similarity between the observed cross-links in the oligomers of the two variants suggests highly conserved solution structures and initial assembly mechanism.

##### Building Atomic Models of ΔN6-β_2_m Oligomers

**FIGURE 4.**
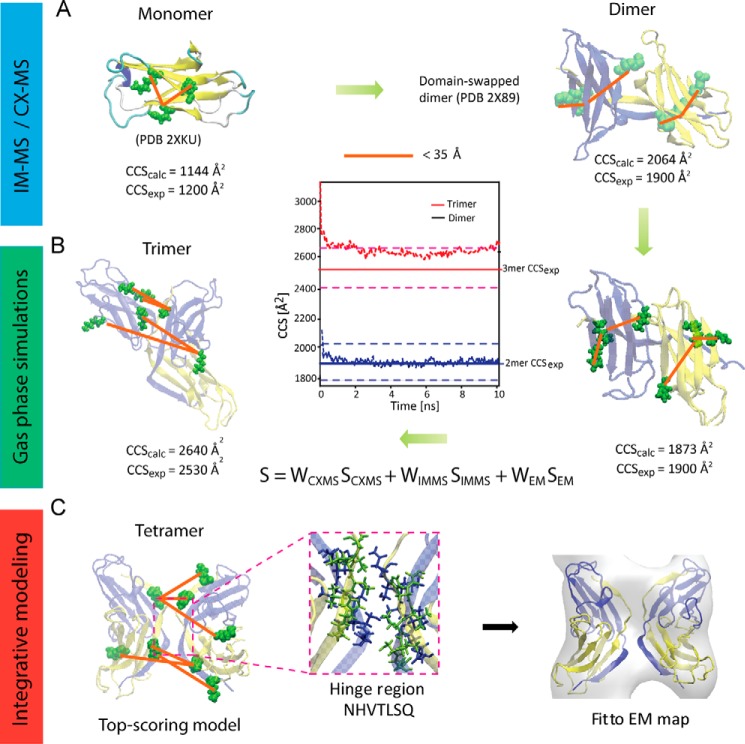
**Workflow for structural characterization of ΔN6-β_2_m tetramer.** *A*, structural data from IM-MS and CX-MS were used to select a suitable starting structure for building the tetramer. The cross-linking data (*orange*) was consistent with ΔN6-β_2_m monomer NMR (PDB ID 2XKU) and dimer x-ray (PDB ID 2X89) crystal structures. Calculated CCS for the energy-minimized monomer was in good agreement with experimental CCS from IM-MS; however, calculated dimer CCS was larger than observed experimentally. *B*, gas-phase MD simulations were performed on the dimer, and the subsequently calculated CCS was in good agreement with experimental CCS, suggesting subtle compaction in the gas phase. Information on the trimer from IM-MS and cross-linking MS were combined using a scoring function in an integrative approach to suggest model structures for trimeric ΔN6-β_2_m, starting from the validated monomer and dimer structures from NMR and x-ray crystallography, respectively. Gas-phase MD simulations were performed on the best-scoring trimeric model structure and CCS calculated, showing good agreement with experimental CCS. *C*, similarly, using restraints from IM-MS, cross-linking MS and EM model structures were suggested for tetrameric ΔN6-β_2_m, starting from the validated dimeric structure (1). Docking of the best-scoring tetramer model into the β_2_m fibril EM density map (EM Database ID 1613) showed excellent agreement, with a cross-correlation coefficient of 0.77.

Having established ΔN6-β_2_m interresidue proximities from CX-MS and the overall compact assembly using IM-MS, we turned our attention to identify suitable atomic resolution structures from the protein data bank (PDB) for building further ΔN6-β_2_m oligomers. NMR structure (PDB ID 2XKU) for ΔN6-β_2_m monomer (50) was used to compare the calculated monomer CCS with that from IM-MS. These were found to differ by 6.3% (CCS_exp_ 1200 ± 36 Å^2^; CCS_calc_ 1276 Å^2^). Rearrangements in the gas phase are thought to be responsible for overall compaction of the structure in the absence of solution leading to lower experimental CCS values than anticipated from the crystal structure (51). To account for this possibility, gas-phase molecular dynamics (MD) simulations were performed before CCS calculations (46). The calculated CCS of the simulated structure was in good agreement with the measured CCS (CCS_exp_ 1200 ± 36 Å^2^; CCS_calc_ 1144 Å^2^; 4.5% deviation). Next, we projected the experimentally identified cross-links (Table 2) onto the atomic structure and measured the Cα-Cα distances (Fig. 4*A*). The calculated distances revealed that the experimentally determined cross-links were in good agreement with the NMR structure using an upper bound interresidue distance threshold of 35 Å (27).

X-ray crystal structure (PDB ID 2X89) is composed of two ΔN6-β_2_m monomers associated through domain-swapping. We compared the calculated CCS from this structure with the measured dimer CCS from IM-MS and found them to differ by 8% (CCS_exp_ 1900 ± 57 Å^2^; CCS_calc_ 2064 Å^2^). Similar to the monomer, we carried out gas-phase MD simulations and found that the calculated CCS of the simulated dimer was in good agreement with the measured CCS from IM-MS (CCS_exp_ 1900 ± 57 Å^2^; CCS_calc_ 1873 Å^2^; 1.4% deviation) (Fig. 4*B*). Structural rearrangement in the gas phase primarily occurred through the compaction of the gross structure as measured by center of mass distances between the two interacting monomers, which decreased from 2.93 to 1.97 nm during the MD simulation. Structural agreement with the experimentally identified cross-links (Table 2) was confirmed by projecting them onto the x-ray crystal structure before and after the MD simulations.

**FIGURE 5.**
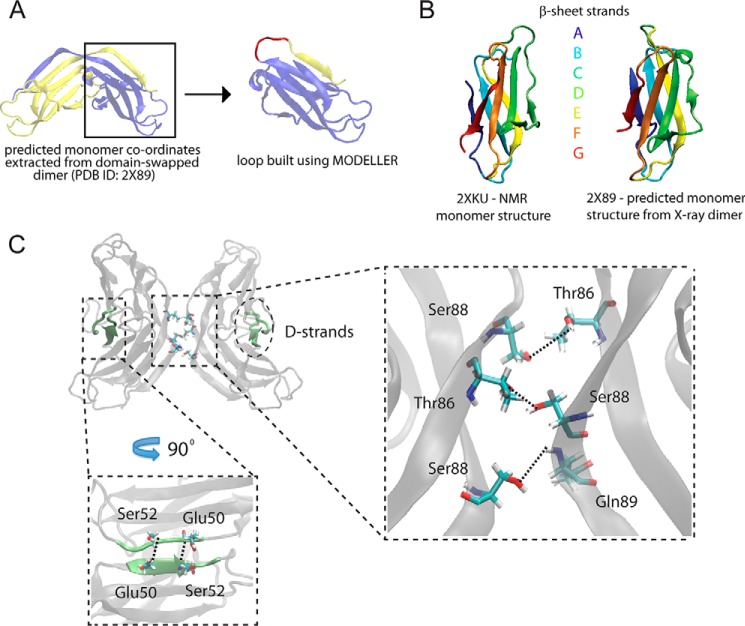
**Starting structures and interaction interfaces for tetrameric ΔN6-β_2_m.** *A*, ΔN6-β_2_m monomer was reconstructed from the domain-swapped dimer x-ray crystal structure (PDB ID 2X89) by extracting the coordinates manually and subsequently rebuilding a missing loop using MODELLER. *B*, the reconstructed monomer shows striking similarity with the monomeric ΔN6-β_2_m structure determined by NMR (PDB ID 2XKU). Colored regions show the corresponding β-sheets A to G in the two structures. *C*, model structure of ΔN6-β_2_m tetramer reveals that intradimeric interfaces are formed by the interaction of the domain-swapped hinge regions and on the opposite side, the D strands interact through hydrogen bonding between Glu-50 and Ser-52. The interdimeric interface is formed from the interaction of the domain-swapped hinge regions by hydrogen bonding of Ser-88 (chain D), Thr-86, and Ser-88 (chain C) with Thr-86 (chain B), Ser-88, and Gln-89 (chain A). Hydrogen bonds were identified using the HBonds tool in VMD (visual molecular dynamics).

Overall we conclude that both the NMR structure for the ΔN6-β_2_m monomer and the x-ray structure for the dimer are consistent with our experimental data and are themselves structurally similar (Fig. 5, *A* and *B*). We, therefore, used these structures as a starting point to build higher order oligomers. Of particular interest is the dimeric structure, as domain-swapping has been proposed as a plausible assembly mechanism for amyloidogenesis (1, 9).

##### Integrative Modeling Predicts ΔN6-β_2_m Oligomers at Atomic Resolution

Having validated starting structures for building higher oligomers, we used an integrative approach to model trimeric and tetrameric ΔN6-β_2_m. To achieve this we computationally integrated our MS data (trimer and tetramer) with available information from EM (tetramer only) using a suitable scoring function (“Experimental Procedures”).

We began by building a model for trimeric ΔN6-β_2_m at atomic resolution. We used as starting structures the domain-swapped dimer and the monomer from NMR. We generated 10,000 atomic models using a Monte Carlo sampling of conformational space, and the models generated were evaluated using the scoring function described above. In particular, we scored all models using the experimentally measured CCS (CCS_exp_ 2530 Å^2^) and four interdimer cross-links, K110:K113 (19.5 Å) K110:K110 (13.4 Å), K110:K67 (29.3 Å), and K110:K27 (18.0 Å), identified in the trimer band (Table 2). Clustering analysis of the top-scoring 1% models revealed two main clusters (threshold 5 Å). We chose a representative structure of the largest cluster (60%). We finally performed gas phase MD simulations (46) showing a compaction (10%; CCS_calc_ 2640 Å^2^) of the trimeric structure in vacuum (Fig. 4*B*), in good agreement with the measured CCS (4% deviation).

Next, we employed a similar strategy to predict the most likely architecture of the tetrameric ΔN6-β_2_m, starting with two dimers (Fig. 4*C*). We scored all models using the experimentally measured CCS (CCS_exp_ 3057 Å^2^), the three identified interdimer cross-links, K110:K113 (22.3 Å), K110:K110 (20.9 Å) and K110:K67 (32.4 Å), for the tetramer (Table 2), and the section of density map corresponding to the globular tetramer of β_2_m (EM Database ID 1613) (11). To reflect the molecular envelope of tetrameric ΔN6-β_2_m, we chose a globular rather than elongated section from the EM map (Fig. 4*C*), consistent with our IM-MS experiments and coarse-grained modeling (Fig. 2*E*). As performed on the trimer, the top 1% scoring models for the tetramer were clustered, and the top scoring model in the largest cluster was chosen as the representative model structure (Fig. 4*C*). Interestingly, in this model the hinge region consisting of heptapeptides NHVTLSQ, which readily form amyloids in isolation, are stacked together (Fig. 5*C*). These interact through dimer-dimer interfaces by hydrogen bonding between Ser-88 (D chain), Thr-86 and Ser-88 (C chain) from one dimer with Thr-86, Ser-88, and Gln-89 from a second dimer, respectively (Fig. 5*C*). Of further interest are the D strands, dynamic regions which are thought to play a role in amyloidogenicity (2, 52). Here, these form the dimer interface on the opposite side to the domain-swapped hinge region (Fig. 5*C*).

**FIGURE 6.**
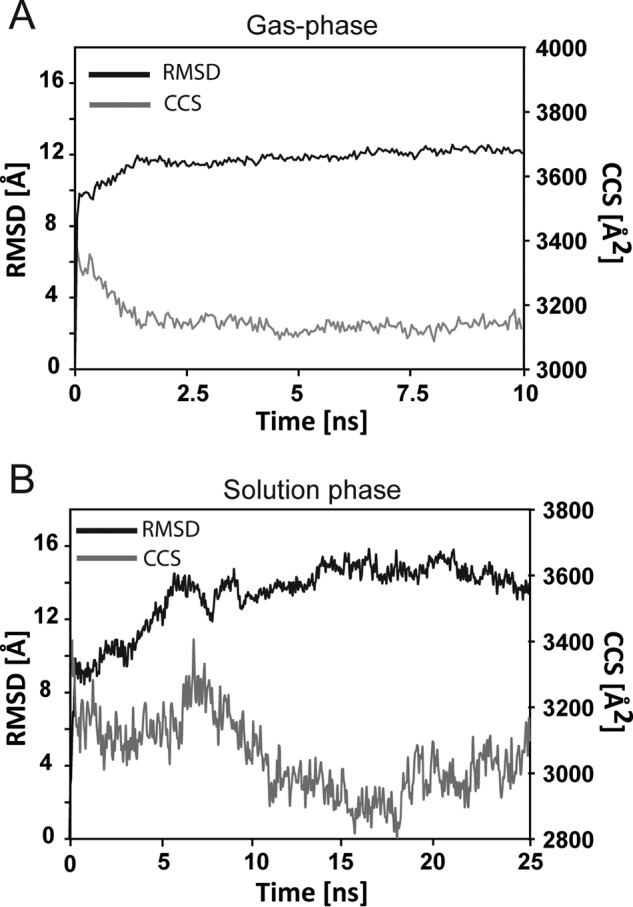
**MD simulations on ΔN6-β_2_m tetramer.** *A*, gas-phase MD simulations of ΔN6-β_2_m tetramer revealed subtle structural rearrangement measured by a decrease in CCS (∼5.9%; *gray line*) over the course of the simulation (10 ns). *B*, solution phase MD simulations of ΔN6-β_2_m tetramer revealed an overall stable conformation as measured by the moderate root mean square deviation (*RMSD*, *black line*) over 25 ns.

Finally, to assess the stability of the predicted tetramer, we performed solution and gas phase MD simulations (46). These simulations revealed that the tetramer underwent only subtle changes over the simulation timeframe, suggesting a stable conformation (Fig. 6, *A* and *B*).

## Discussion

ΔN6-β_2_m oligomers have been proposed as intermediate assemblies leading to fibrillogenesis either through nucleation and elongation of their own fibrils or through cross-seeding with full-length β_2_m (2). β_2_m fibrillogenesis may be seeded by preformed ΔN6-β_2_m filaments or fibrils (53). Alternatively, ΔN6-β_2_m monomers may interact with full-length β_2_m, enabling a transition into an aggregation-prone conformation (50). Evidence in support of this, from NMR (50) and IM-MS (18), shows that β_2_m monomers undergo a conformational change before oligomerization. This intermediate state is thought to have enhanced amyloidogenic potential (2). A similar intermediate state may be more readily accessible for ΔN6-β_2_m, giving rise to its increased amyloidogenicity compared with full-length β_2_m (2, 54, 55). Another possibility is that a proteolytic step may precede aggregation, in which the N-terminal hexapeptide is removed from β_2_m and that ΔN6-β_2_m is, therefore, itself an on-pathway intermediate of β_2_m fibrillation (54). Although the specific interaction between β_2_m and ΔN6-β_2_m remains a topic of intense debate, it is becoming increasingly clear that studying the early oligomeric pathway of ΔN6-β_2_m at atomic level may be essential to understand the assembly mechanism for full-length β_2_m.

Here we predict structural models and an early assembly mechanism for oligomers of ΔN6-β_2_m up to tetramer, highlighting their compact topologies and key intersubunit interactions and comparing oligomers of the full-length protein. Because fibrillogenesis has been observed for ΔN6-β_2_m under similar solution conditions to those used in our MS experiments (1), we believe that the observed oligomers serve as pre-amyloid intermediates en route to higher order oligomeric species and fibril formation.

From our comparative IM-MS and CX-MS results, we established that ΔN6-β_2_m and full-length β_2_m had similar oligomer profiles, CCS, and identified cross-links. This suggests a high structural similarity for the lower order oligomers of these two variants and points to a conserved initial assembly mechanism before fibrillogenesis. The specific experimental conditions, however, are important for determining the *ex vivo* aggregation pathway followed and resulting fibril morphology (18, 19, 56). Previous studies of full-length β_2_m have shown that at low pH and low ionic strength, long straight fibrils are formed via a nucleated mechanism. On the other hand, at higher pH and ionic strength, worm-like fibrils are formed, with elongation proceeding via a non-nucleated, monomer addition pathway, and a conformational change in monomeric β_2_m thought to be responsible for initiating oligomerization. Interestingly, assembly of β_2_m in the presence of Cu(II) ions proceeds through dimer addition (52), with domain-swapping also implicated (57).

**FIGURE 7.**
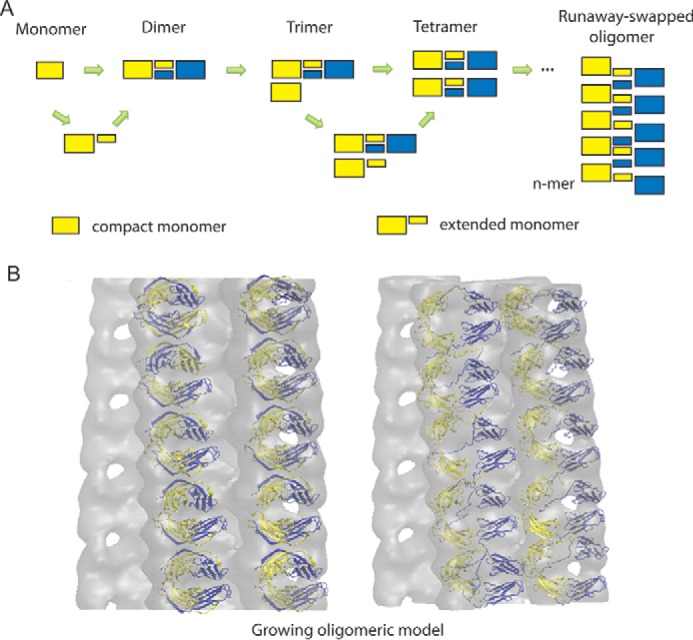
**Mechanistic models for growing ΔN6-β_2_m oligomers.** *A*, proposed assembly mechanism of early oligomers preceding amyloidosis. A stepwise monomeric addition is suggested for oligomerization that likely involves an extended “open” intermediate state before domain swapping. *B*, Multiple copies of tetrameric ΔN6-β_2_m model were fitted into the β_2_m fibrillar EM map (EM Database ID 1613). The growing oligomer models were built from repeating tetramers, where the domain-swapped hinge region was perpendicular to the fibrillar growth axis (*left*), consistent with the transition of stacked domain-swapped dimers into runaway oligomers (*right*).

Although the precise assembly mechanism(s) followed *in vivo* are not fully understood, the evidence is growing for a domain-swapped oligomeric intermediate preceding fibril formation (1, 9). By exploiting the power of MS, we put forward a model at the atomic level of resolution, describing the early formation of ΔN6-β_2_m oligomers, indicating a stepwise assembly mechanism through the addition of monomeric subunits (Fig. 7*A*). We further mapped the oligomeric growth into the fibrillar EM map to show the conversion of stacked domain- to runaway-swapped oligomers in which the ends of the growing oligomers are capable of binding open monomers by self-templated growth (Fig. 7*B*). Overall, this study provides novel insights into the early mechanism of pre-amyloid assemblies and further makes inroads toward an atomic level description of β_2_m that entails detailed structural models of intermediate states and their associated transient interactions.

## Author Contributions

Z. H. and A. P. conceived and designed the study. Z. H. conducted all native MS experiments. C. S. conducted all cross-linking experiments. Z. H. and A. P. performed the modeling. Z. H., C. S., and A. P. analyzed the data and wrote the paper.

## Footnotes

2 The abbreviations used are:

β_2_m: β_2_-microglobulin
BS3: bis[sulfosuccinimidyl] suberate
CCS: collision cross-section
CX: cross-linking
ΔN6-β_2_m: truncated β_2_-microglobulin variant lacking six N-terminal residues
IM: ion mobility
PA: projection approximation
MD: molecular dynamics.
